# Evaluating the Performance of 3D-Printed Stab-Resistant Body Armor Using the Taguchi Method and Artificial Neural Networks

**DOI:** 10.3390/polym17192699

**Published:** 2025-10-07

**Authors:** Umur Cicek

**Affiliations:** Design School, Loughborough University, Loughborough LE11 3TU, UK; u.i.cicek@lboro.ac.uk

**Keywords:** 3D-printed body armor, stab resistance, impact, Taguchi method, artificial neural networks, HOSDB

## Abstract

Additive manufacturing has promising potential for the development of 3D-printed protective structures such as stab-resistant body armor. However, no research to date has examined the impact of 3D printing parameters on the protective performance of such 3D-printed structures manufactured using fused filament fabrication technology. This study, therefore, investigates the effects of five key printing parameters: layer thickness, print speed, print temperature, infill density (Id), and layer width, on the mechanical and protective performance of 3D-printed polycarbonate (PC) armor. A Taguchi L_27_ matrix was employed to systematically analyze these parameters, with toughness, stab penetration depth, and armor panel weight as the primary responses. ANOVA results, along with the Taguchi approach, demonstrated that Id was the most influential factor across all print parameters. This is because a higher Id led to denser structures, reduced voids and porosities, and enhanced energy absorption, significantly increasing toughness while reducing penetration depth. Morphological analysis supported the statistical findings regarding the role of Id on the performance of such structures. With optimized printing parameters, no penetration to the armor panels was recorded, outperforming the UK body armor standard of a maximum permitted knife penetration depth of 8 mm. Moreover, an artificial neural network (ANN) utilizing the 5-14-12-3 topology was created to predict the toughness, stab penetration depth, and armor panel weight of 3D-printed armors. The ANN model demonstrated better prediction performance for stab penetration depth compared to the Taguchi method, confirming the successful application of such an approach. These findings provide a critical foundation for the development of high-performance 3D-printed protective structures.

## 1. Introduction

Additive manufacturing (AM) is defined as the process of joining materials to fabricate parts directly from 3D model data, typically layer upon layer, in contrast to subtractive and formative manufacturing technologies. According to the American Society for Testing and Materials (ASTM), AM is categorized into seven primary technologies [1]: vat photopolymerization, material jetting, binder jetting, sheet lamination, directed energy deposition, powder bed fusion (PBF), and material extrusion (MEX). With the growing adoption of AM, its applications have expanded across various fields, including electronics [2,3,4], defense [5], aerospace [6], and biomedical [7]. Among various AM applications, 3D-printed textiles have garnered increasing attention due to their unique properties, such as flexibility, drapability, and wearable mechanical performance, as well as their potential for clean and sustainable manufacture [8,9]. Bingham et al. were the first to highlight the design and manufacturing possibilities of 3D-printed textiles using AM techniques [10]. For the fabrication of such textiles, PBF and MEX technologies are commonly employed [11,12]. Although primarily used for fashion applications today [8,11], the design flexibility and promising material properties of AM have also enabled researchers to develop engineering-grade 3D-printed protective textiles such as 3D-printed stab-resistant body armor (SRBA) [13,14]. Such protective structures have the potential to be a viable alternative to conventional body armor by addressing key drawbacks, including excessive weight, poor thermal and moisture management, and restricted mobility and flexibility.

A significant part of existing research on 3D-printed SRBA has focused on the use of selective laser sintering (SLS), a PBF technology. Various designs of armor specimens manufactured via SLS have been evaluated following the body armor standards of the UK Home Office Scientific Development Branch (HOSDB) [15]. These include single-layer planar specimens fabricated from polyamide (PA) 12 [16], dual-layered planar specimens from PA 12 [17], and both planar and articulated scale-like specimens from PA 2200 [13]. Different SLS materials have also been employed for the development of 3D-printed armor. For example, multi-layered planar specimens composed of PA 3200 and PA 4300, as well as pyramid-structured plates from PA 3200, have been analyzed using finite element analysis (FEA) and validated for their stab protection [18]. Moreover, single and dual-layered planar and pyramid-like samples made from PA 3200 have been assessed for their stab protective performance [19,20], as well as the impact of utilizing carbon fiber in armor samples [21]. Moreover, bio-inspired designs have been demonstrated as an alternative to planar armor scales: imbricated scales made from PA 12 [22] and egg-shelled structures made from PA 3200 [23].

Moreover, fused filament fabrication (FFF), a MEX technology, has been used for the fabrication of SRBA. The stab resistance performance of 3D-printed body armor manufactured from pure and polycarbonate (PC)-blended acrylonitrile butadiene styrene (ABS) was estimated using FEA and validated through a series of stab tests [24,25]. In addition, aramid fiber-reinforced armor test specimens manufactured at different thicknesses using FFF were stab-tested [26]. FFF-manufactured polylactic acid (PLA) armor panels with different infill densities and types, of which internal voids were filled with epoxy resin to create composite structures, were subsequently stab tested [14,27]. More recently, using FFF technology, Cicek et al. printed square armor scales with lateral dimensions ranging from 40 mm to 80 mm and thicknesses between 6 mm and 10 mm using various materials. The specimens were tested according to the HOSDB KR1-E1 standard [15], where PC exhibited the highest stab resistance, requiring only a 5 mm thickness for effective protection [14].

In terms of MEX, PC has appeared to be a promising candidate for 3D-printed SRBA in the previous research [14]. Given that 3D-printing process parameters play a crucial role in the mechanical performance of the 3D-printed parts, optimizing these is essential to enhance both the mechanical and protective performance of 3D-printed SRBA. For example, Mohamed et al. examined six 3D print variables, namely layer thickness, air gap, raster angle, build orientation, line width, and the number of contours, to evaluate their impact on print time, material usage, and mechanical behavior in PC/ABS composites [28]. In a related study, Masood et al. explored how air gap, raster angle, and line width, each tested at three levels, influenced the tensile strength of PC components [29]. Vidakis et al. investigated the influence of three levels of layer thickness and nozzle temperature on the strain rate sensitivity of PC samples fabricated using FFF, testing them at five elongation speeds [30]. Subsequently, an L_9_ full-factorial matrix was develop to optimize the levels of layer thickness and nozzle temperature, with the aim of improving the mechanical properties of PC-based parts [31]. The Taguchi method has also become a prevalent tool for optimizing process parameters in FFF-printed parts [32]. One study utilized an L_27_ Taguchi orthogonal array to explore the influence of seven parameters: infill density, raster angle, nozzle temperature, print speed, layer thickness, bed temperature, and orientation angle, focusing on energy consumption and compressive properties of PC parts [33]. Similarly, Verma et al. implemented an L_27_ Taguchi design to assess how print temperature, bed temperature, print speed, number of shells, layer thickness, and infill ratio affect the ultimate tensile strength of PC/ABS printed parts [34]. Srinivas et al. conducted a study utilizing an L_9_ Taguchi design to examine how infill ratio, layer thickness, and print speed affect the mechanical behavior, specifically tensile, compressive, and flexural properties, of 3D-printed PC components [35]. In a more recent investigation, Cicek and Johnson adopted an L_27_ Taguchi matrix to evaluate the impact of five 3D-printing parameters, namely layer thickness, print speed, print temperature, infill density, and line width, on the tensile strength, material consumption, and build time of 3D-printed PC parts. To further optimize the process, gray relational analysis was applied, aiming to enhance tensile strength with minimal material usage and production time [36].

Moreover, there has been a recent shift toward employing machine learning (ML) techniques for performance prediction based on the print parameters of 3D-printed parts, rather than relying solely on traditional statistical tools. This trend is driven by the highly non-linear relationships between inputs (print parameters) and outputs (performance parameters) in 3D printing, where conventional statistical methods may be insufficient for accurate prediction. ML techniques offer a powerful alternative by enabling the construction of predictive models that can simultaneously manage multiple process and material parameters while capturing complex, non-linear interactions between inputs and outputs [37]. Among various ML approaches, artificial neural networks (ANNs) have emerged as one of the most widely used techniques in the field of AM, owing to their ability to learn from experimental data and accurately characterize non-linear and interaction effects [38,39]. Several studies have demonstrated the effectiveness of ANN models in predicting various performance characteristics of 3D-printed parts across different materials and processing parameters. Contuzzi et al. utilized response surface methodology (RSM) to train an ANN architecture for predicting the geometry and density of PLA/wood biocomposites based on three print parameters, finding that the ANN model outperformed traditional regression approaches [40]. Boppana and Ali investigated the influence of four print parameters on the mechanical properties of 3D-printed PC parts, where the ANN model demonstrated superior prediction accuracy compared to regression methods [41]. Saad et al. developed an ANN model to predict the surface roughness of 3D-printed PLA components based on four printing parameters, achieving a 12.36% improvement in performance over the RSM approach [42]. Similarly, Giri et al. employed an ANN framework to predict tensile strength, build time, and surface roughness of PLA prints using six different print parameters, reporting a high correlation coefficient between experimental and predicted values [43]. Sood et al. applied an ANN architecture to estimate the compressive properties of ABS 3D-printed parts based on five printing parameters, observing negligible variation between the model predictions and experimental measurements [44]. Borah and Chandrasekara predicted surface roughness, mechanical strength, and elastic modulus of 3D-printed polyetheretherketone parts using four print parameters, with the developed ANN achieving an average prediction error of less than 5% compared to experimental results [45].

Despite extensive research on the mechanical properties of 3D-printed PC, the influence of print parameters on its stab resistance remains largely unexplored. Understanding and predicting this relationship is critical for optimizing the protective performance and comfort of additively manufactured structures, particularly as interest in 3D-printed SRBA continues to grow. Prior to performance optimization, it is essential to first establish how print parameters affect protective capabilities. Therefore, the present study investigates the effects of 3D printing parameters on the mechanical and stab resistance properties of 3D-printed PC body armor. To achieve this, a Taguchi L_27_ design was developed, and the Taguchi method along with Analysis of Variance (ANOVA) were employed to analyze the effects of these parameters on toughness, stab penetration depth, and armor panel weight. Additionally, an ANN model was developed to predict these properties. Data from the L_27_ Taguchi array were used to train, validate, and test the ANN, and the results were compared with those from the Taguchi method and experimental tests.

## 2. Materials and Methods

### 2.1. Test Samples

**Figure 1:**
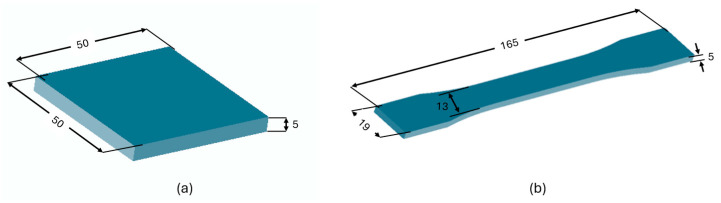
Dimensions of test specimens. (**a**) armor panel and (**b**) tensile. All dimensions are in mm.

Two types of test samples were designed: a tensile test specimen and a body armor panel, using SolidWorks^®^ (Dassault Systemes, Waltham, MA, USA). While the design of the tensile test specimen was in compliance with the Type I of ASTM D638: Standard Test Method for Tensile Properties of Plastics [46], the geometry and dimensions of the body armor panel were selected based on existing literature [14]. The dimensions of the test samples are illustrated in Figure 1.

Due to its high impact resistance and previous successful use in 3D-printed armor manufacturing, PC was selected for manufacturing the test specimens, specifically using Ultimaker’s 2.85 mm diameter PC filament. The process parameters were employed based on the literature survey and previous research [36] and include layer thickness (Lt), print speed (Ps), print temperature (Pt), infill density (Id), and layer width (Lw). When selecting the print parameters, industrially relevant extremes for PC-based armor manufacturing were considered. Therefore, Lt and Lw levels were chosen based on the most reported values in the literature, while Ps and Pt were set according to the filament manufacturer’s specifications to ensure adequate flow without material degradation and compatibility with the printer. The lower limit of Id was defined to maintain the strength required in armor applications, where high structural integrity is essential. A summary of all printing parameters utilized in this study is provided in Table 1.

The Taguchi method was employed to examine the influence of selected variable printing parameters on the investigated responses: toughness, stab penetration depth, and armor panel weight. By utilizing five variable printing parameters, each at three levels, an L_27_ Taguchi matrix was developed, as summarized in Table 2. This method significantly reduced the required number of specimens from 243 to 27 per sample, with five replications for each condition, totaling 135 tensile specimens and 135 armor panels.

Ultimaker Cura (version 4.9) was used to generate g-codes based on the fixed and variable parameters, following the sequence defined in the Taguchi L_27_ orthogonal array. For each set of parameters, two test specimens, a body armor panel and its corresponding tensile specimen, were fabricated in the same production run on an Ultimaker^3^ 3D printer (Ultimaker BV, Utrecht, The Netherlands).

### 2.2. Experimental Procedure

Tensile tests were performed on an Instron 3366 universal testing machine (Instron, Norwood, MA, USA), equipped with a 10 kN load cell and a 25 mm gauge length extensometer, at a crosshead speed of 5 mm/min, in accordance with the ASTM D638 standard requirement for Type I specimens. Tensile data was recorded via Instron’s Bluehill Universal software (version 4.18). Average values for toughness were derived from the stress–strain data as the area under the stress–strain curve using a MATLAB script that applied the trapezoidal rule.

Stab testing was conducted to assess the penetration resistance of the 3D-printed armor panels against stab impact loads that could cause sudden deformation or complete rupture. The stab tests were carried out according to the UK HOSDB KR1-E1 standard, which specifies a 24 J energy level for knife resistance [15]. This level is widely accepted as representative of common stabbing threats and therefore serves as a baseline for comparison with higher threat levels involving greater impact energies. The tests were performed using an HOSDB gravity-driven drop tube assembly. The test apparatus consisted of a dropped hammer fitted with an HOSDB P1/B test knife and a composite backing material designed to replicate the compressible surface of the human body. The hammer was released from a predetermined height to deliver the required impact energy, striking the 3D-printed armor panel positioned on the backing material. Upon impact, stab penetration depth, a critical measure of stab resistance, was measured using a digital caliper (with a readability of 0.01 mm) on the underside of each specimen at the knife’s tip. Additionally, the weight of the fabricated armor panels was measured using a scale (Model: LE6220S, Sartorius, Göttingen, Germany), having a readability of 0.01 g to ensure accurate weight determination.

### 2.3. Statistical Analysis

The experiments were conducted according to the sequence defined by the L_27_ Taguchi matrix, and the resulting data were statistically analyzed using Minitab 20.3 software. The individual effects of each printing parameters on investigated responses were assessed by analyzing signal-to-noise (SN) ratios. The SN ratios were calculated using the larger-the-better (LtB) criterion for toughness, and the smaller-the-better (StB) criterion for both stab penetration depth and armor panel weight. Regardless of the criterion applied, a higher SN signifies better quality characteristics, indicating parameter settings that effectively reduce the influence of noise. Equations (1) and (2) were used to calculate SN ratios for the LtB and StB approaches, respectively.

(1) $$SN=-10log\left[\frac{1}{n}\sum_{i=1}^{n}\frac{1}{Y_{i}^{2}}\right]$$

(2) $$SN=-10log\left[\frac{1}{n}\sum_{i=1}^{n}Y_{i}^{2}\right]$$

Here, n denotes the number of experiments conducted, and Y_i_ is the outcome of the ith experiment.

ANOVA was used to determine and signify the individual percentage contribution of each printing parameters to the investigated outcomes. Additionally, Pearson’s correlation analysis was utilized on Minitab software to explore the interrelated relationships between the selected responses. Correlations were categorized according to the absolute value of the correlation coefficient (|*r*|): small (0.1 ≤ |*r*| < 0.3), moderate (0.3 ≤ |*r*| < 0.5), and strong (|*r*| > 0.5).

Moreover, a morphological investigation of the tensile-tested specimens was conducted using scanning electron microscopy (SEM) on a GeminiSEM 300 (Carl Zeiss, Oberkochen, Germany), focusing on their fractured surfaces. To prepare the specimens, a thin layer of gold was sputter-coated using a rotary-pumped coater (Q150R S, Quorum, East Sussex, UK). The gold-coated specimens were then placed in the microscope, and SEM imaging was performed at an accelerating voltage of 5.00 kV.

### 2.4. ANN Model

Although alternative ML approaches are often effective for small datasets, this study focused on the ANN due to its widespread use in the AM literature and its ability to flexibly approximate non-linear relationships, as is often the case in 3D printing. Data from the L_27_ Taguchi array were used to create the ANN using MATLAB 2024b, consisting of three layers: input, hidden, and output. The input layer included five neurons representing the selected 3D printing parameters: Lt, Ps, Pt, Id, and Lw. The output layer had three neurons corresponding to the investigated properties: toughness, stab penetration depth, and armor panel weight. The number of hidden layers and neurons was optimized using a trial-and-error approach to achieve the best predictive performance. A MATLAB script was developed to automatically test different network configurations, ranging from one to three hidden layers. The script evaluated networks with one, two, or three hidden layers, with the number of neurons in each layer varying between 1 and 20. The performance of each developed ANN model was then assessed using the mean squared error (MSE) and the coefficient of determination (R^2^), with lower MSE and higher R^2^ indicating better prediction accuracy for the three output properties. Therefore, the architecture yielding the lowest MSE and highest R^2^ was selected as the optimal ANN model for further analysis.

The data from the L_27_ Taguchi matrix were then used to model the ANN, which were divided into three subsets: 70% for training, 15% for validation, and 15% for testing. The Levenberg–Marquardt algorithm (trainlm) was used for training, and the tangent sigmoid function was applied for the transfer function of the hidden neurons. Furthermore, to mitigate the risk of overfitting with a limited dataset, training was conducted with a small learning rate of 0.001 to ensure stable convergence, with early stopping applied based on validation error of 10 checks, a regularization factor of 0.02 to penalize overly complex weights, and a maximum of 1000 epochs set as the upper training limit.

## 3. Results and Discussion

### 3.1. Experimental and Statistical Investigation

**Figure 2:**
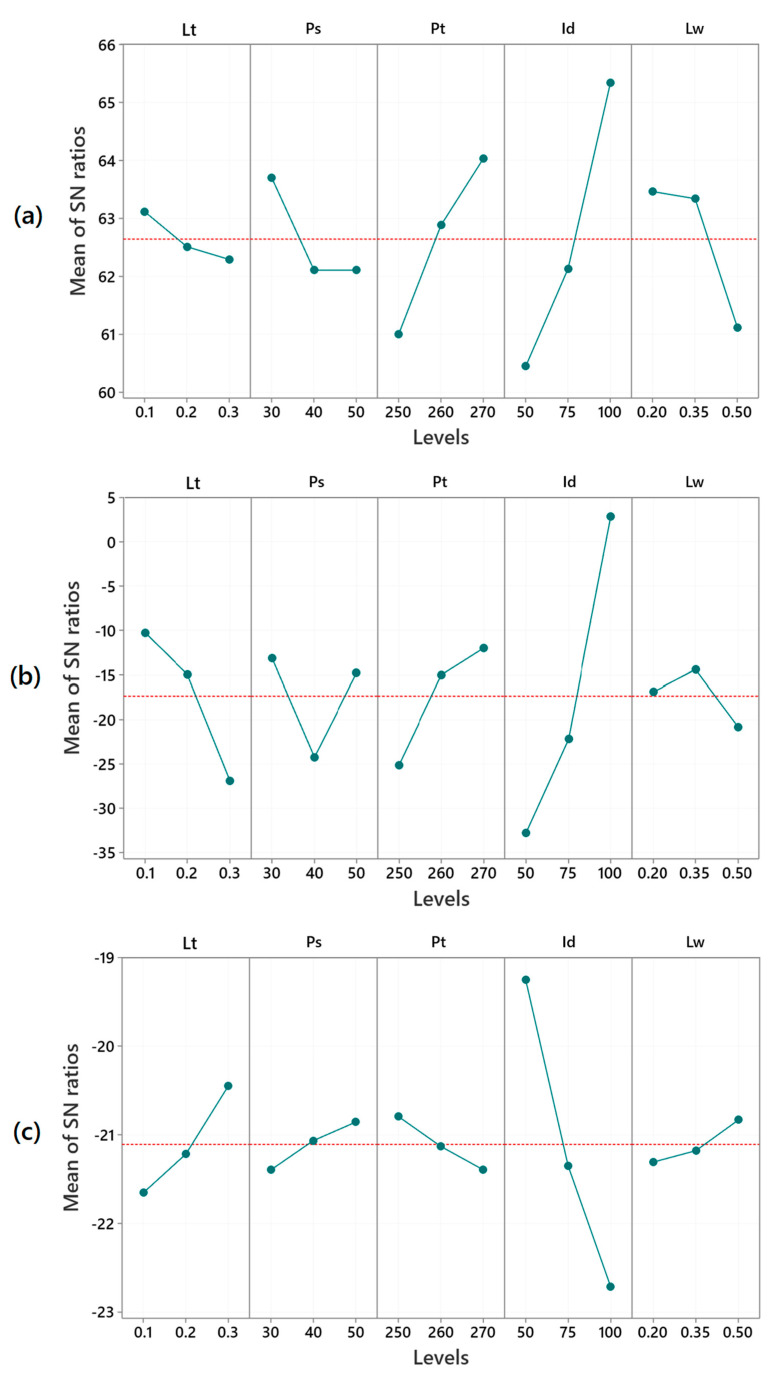
Mean SN ratios for (**a**) toughness, (**b**) stab penetration depth, and (**c**) armor panel weight.

This study examines the influence of five variable 3D printing parameters on the protective performance of PC samples. The SN ratios are detailed in Table 3 and illustrated in Figure 2. Moreover, the data used in this study can be found in the table provided in Supplementary Table S1. According to results, specimen 7 exhibited the highest toughness (2418.25 kJ/m^3^) and the best stab protective performance, as so the lowest stab penetration depth of only 0.02 mm. In contrast, specimen 27 had the lightest armor panel weight at 7.67 g.

The detailed results of the Taguchi analysis for toughness are summarized in Table 4. Higher toughness values were observed at lower levels of Lt and Lw, while higher levels of Pt and Id had a positive impact on toughness. However, setting Ps at 40 or 50 mm/s had no effect on toughness. The optimal printing parameters for the highest toughness was identified as Lt at 0.1 mm, Ps at 30 mm/min, Pt at 270 °C, Id at 100%, and Lw at 0.2 mm, or shortly Lt_1_Ps_1_Pt_3_Id_3_Lw_1_.

As shown in Table 5, the results of ANOVA indicate that all parameters, except Lt, had a statistically significant effect on toughness, as evidenced by their *p*-values below 0.05. The mathematical model used to predict the toughness of specimens is given in Equation (3). The model also demonstrated high precision with an R^2^ value of 93.92%.

Toughness (kJ/m^3^) = 1484.5 + 61.5Lt_0.1 − 13.4Lt_0.2 − 48.1Lt_0.3 + 189.2Ps_30 − 154.4Ps_40 − 34.8Ps_50 − 306.6Pt_250 + 53.5Pt_260 + 253.2Pt_270 − 374.7Id_50 − 115.8Id_75 + 490.5Id_100 + 134.3Lw_0.20 + 98.7Lw_0.35 − 233.0Lw_0.50 (3)

**Figure 3:**
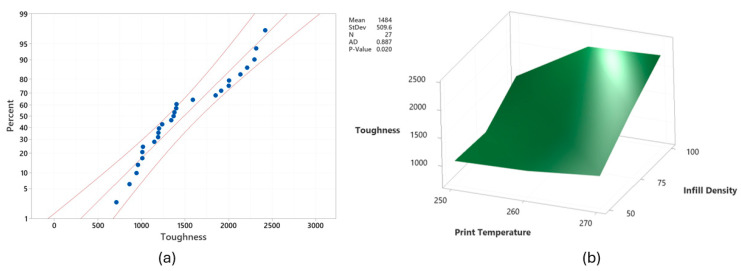
(**a**) probability plot for toughness and (**b**) effect of infill density and print temperature on toughness when Lt 0.1 mm, Ps 30 mm/s, and Lw 0.20 mm.

Moreover, as seen in Figure 3a, the data were mostly within the probability limits. The ANOVA findings aligned with the Taguchi analysis results. Among the process parameters, Id was the most influential, contributing 52.58% to the toughness, followed by Pt at 21.46%. This is likely because higher Id led to denser structures with fewer voids, while elevated Pt improved layer adhesion, collectively enhancing the toughness of the 3D-printed parts. This relationship is further demonstrated in Figure 3b. These findings align with the literature, where increased Id reduces void formation, and higher Pt strengthens interlayer bonding, improving mechanical integrity and toughness [33,47,48,49]. Additionally, Lw and Ps exhibited notable contributions of 10.94% and 8.11%, respectively. However, Lt had a minimal effect on toughness, contributing only 0.84%. Given that toughness is highly dependent on bonding quality and structural integrity, variations in Lt within the tested range likely did not significantly affect layer adhesion or stress distribution, both of which are critical to toughness [50].

Since the optimal printing parameters for maximizing toughness were not included in the initial L_27_ design, a new set of tensile specimens was fabricated using the optimal printing parameters and tested. These specimens achieved a toughness value of 2664.39 kJ/m^3^, reflecting an approximately 10% improvement over the highest values recorded for specimen 7.

**Figure 4:**
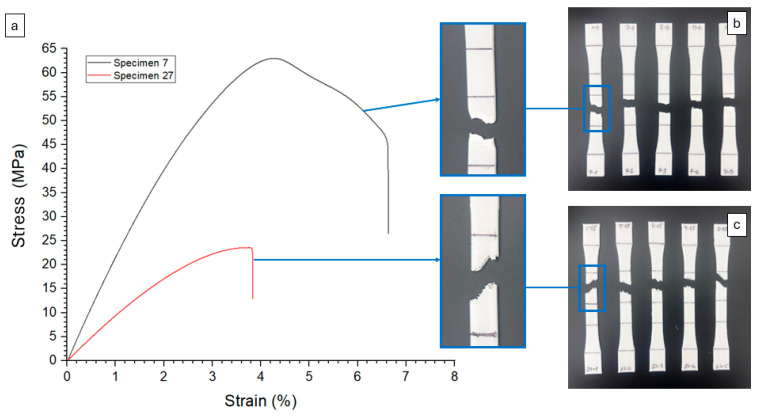
(**a**) stress–strain behavior of selected specimens, and fractured surfaces of (**b**) specimen 7 and (**c**) specimen 27.

Figure 4 compares the mechanical behavior of specimens 7 and 27, providing insights into their stress–strain characteristics (Figure 4a) and fracture surfaces. Specimens with higher Id exhibited more ductile fractures, as seen with specimen 7, which had the highest toughness. As seen in Figure 4b, its fracture surface displayed uniform plastic deformation (as also witnessed from the necking in specimen 7), suggesting a well-distributed internal structure that allowed for substantial elongation before failure. In contrast, specimen 27 exhibited brittle fracture behavior, with sharper and irregular breakage patterns as demonstrated in Figure 4c. The abrupt failure and lack of plastic deformation indicated weaker interlayer bonding or a more fragile internal structure, likely leading to premature crack propagation and sudden failure. These differences highlight the critical role of Id in the mechanical performance of 3D-printed parts. The findings suggest that optimizing printing parameters can enhance toughness and reliability, particularly for applications requiring durability and resistance to mechanical stress.

Table 6 presents the detailed SN ratios for stab impact protection based on the Taguchi results. The optimal printing parameters, identified by the minimum stab penetration, were Lt_1_Ps_1_Pt_3_Id_3_Lw_2_, corresponding to Lt: 0.1 mm, Ps: 30 mm/s, Pt: 270 °C, Id: 100%, and Lw: 0.35 mm. Further, higher levels of Pt and Id improved stab protection, while reducing Lt had a similar effect. For Ps, levels 1 and 3 yielded comparable results, whereas level 2 had the least impact. Regarding Lw, printing at level 2 provided the highest stab protection.

The ANOVA results for stab impact protection are presented in Table 7, with an R^2^ value of 89%, indicating an acceptable level of accuracy. Moreover, the mathematical model used to predict the stab penetration depth of specimens is given in Equation (4).

Stab penetration depth (mm) = 22.9 − 8.6Lt_0.1 − 1.7Lt_0.2 + 10.3Lt_0.3 − 8.45Ps_30 + 0.27Ps_40 + 8.2Ps_50 − 2.4Pt_250 + 6.4Pt_260 − 4.0Pt_270 + 26.16Id_50 − 7.9Id_75 − 18.3Id_100 + 7.0Lw_0.20 − 5.2Lw_0.35 − 1.8Lw_0.50(4)

**Figure 5:**
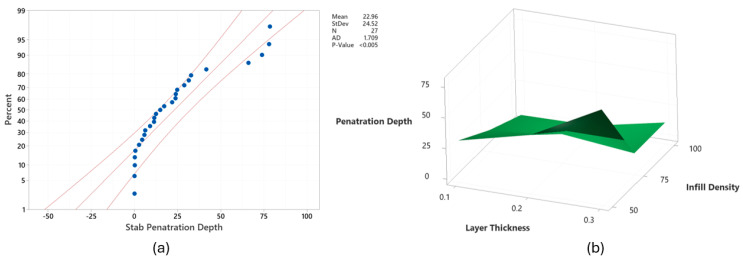
(**a**) probability plot for stab penetration depth and (**b**) effect of infill density and layer thickness on stab penetration depth when Ps 30 mm/s, Pt 270 °C, and Lw 0.35 mm.

Figure 5a shows the probability plot for stab penetration depth, with the data predominantly falling within the probability limits, exhibiting a moderate level of scatter around the fitted line. The findings indicated that Lt, Ps, and Id were statistically significant factors with contributions of 10.52%, 7.96%, and 62.20%, respectively. The Taguchi and ANOVA results have shown that Id was the most important factor for the stab protection capability of 3D-printed textiles. This finding is comparable to the existing literature [49,51,52,53,54,55]. This is because increasing Id reduces voids and porosity by increasing material density, which, in turn, enhances impact resistance as more material absorbs and distributes the impact force. Being the two most influential print parameters, the effect of Lt and Id on stab penetration depth is demonstrated in Figure 5b.

**Figure 6:**
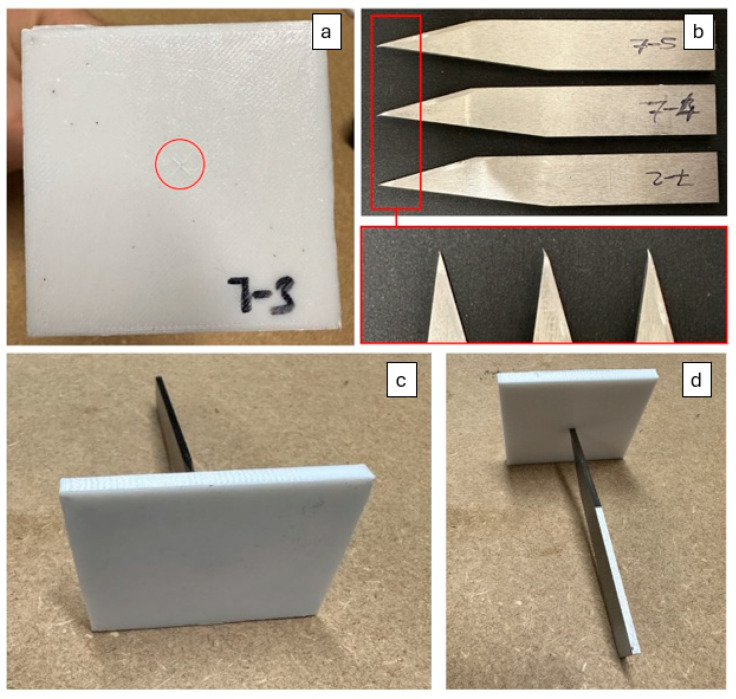
(**a**) specimen 7 after stab testing showing minimal penetration, (**b**) damage observed on the knife tips, and (**c**,**d**) front and back views of the optimized armor panel, showing no recorded stab penetration.

The results showed that specimen 7 had the lowest mean stab penetration depth of 0.02 mm, which is barely visible as seen in Figure 6a. This performance not only surpasses the UK HOSDB standard threshold of 8 mm [15] but also far exceeds the requirements of the US National Institute of Justice standard for stab-resistant armor, which specifies a maximum allowable penetration threshold of 7 mm for Level 1 protection [56], thereby demonstrating exceptional robustness and broad international relevance. Their effectiveness was further demonstrated by damage to the knife tips used in testing (Figure 6b).

Moreover, a new set of armor panels, fabricated using optimized print parameters, exhibited no penetration (Figure 6c,d). However, this improvement resulted in an 8.25% increase in 3D-printed armor panel weight compared to specimen 7. Although this additional weight is relatively modest, it may influence wearer comfort, especially during extended use, where factors such as mobility and fatigue are critical. In practical applications, even small increases in armor weight can affect operational performance. This underscores the importance of not only maximizing protective performance (e.g., minimizing penetration depth) but also ensuring ergonomic suitability (e.g., minimizing armor weight) when designing 3D-printed stab-resistant armor.

Throughout history, the two major requirements of body armor, protection and comfort, have always been in conflict [57]. Research confirms that the torso region that body armor covers is particularly vulnerable due to its role in shielding vital organs, such as the heart and lungs [58]. Among the factors influencing wearer comfort, armor weight is the most critical, necessitating a systematic investigation into how 3D printing parameters affect panel weight optimization. The detailed SN ratios for armor panel weight are presented in Table 8. Increases in Lt, Ps, and Lw reduced armor panel weight, while decreasing Pt and Id led to lighter panels. The optimal parameter set for minimizing armor panel weight was identified as Lt_3_Ps_3_Pt_1_Id_1_Lw_3_, corresponding to Lt: 0.3 mm, Ps: 50 mm/min, Pt: 250 °C, Id: 50%, and Lw: 0.5 mm.

**Figure 7:**
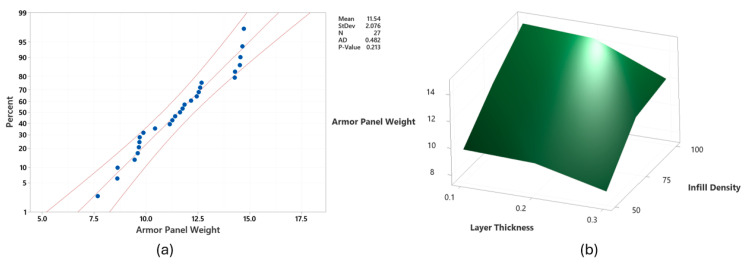
(**a**) probability plot for armor panel weight and (**b**) effect of infill density and layer thickness on armor panel weight when Ps 50 mm/s, Pt 250 °C, and Lw 0.50 mm.

As seen in Figure 7a, the data regarding armor panel weight was in the probability limits with a few exceptions. The ANOVA model, presented in Table 9, demonstrated high statistical precision with an R^2^ value of 98.99%. Furthermore, the mathematical model used to predict the armor panel weight of specimens is given in Equation (5).

Armor panel weight (g) = 11.55 + 0.73Lt _0.1 + 0.14Lt_0.2 − 0.86Lt_0.3 + 0.36Ps_30 − 0.15Ps_40 − 0.2Ps_50 − 0.53Pt_250 + 0.15Pt_260 + 0.39Pt_270 − 2.34Id_50 + 0.16Id_75 + 2.18Id_100 + 0.25Lw_0.20 + 0.09Lw_0.35 − 0.34Lw_0.50(5)

All printing parameters had a statistically significant impact on armor panel weight, as indicated by *p*-values less than 0.05. Among them, Id was identified as the most influential factor, contributing 82.17%, followed by Lt with a smaller effect of 10.21%. The relationship between Id and Lt in relation to armor panel weight is demonstrated in Figure 7b. However, the remaining parameters had minimal impact on armor panel weight. A new set of armor panels using optimal printing parameters for weight reduction was fabricated, but not stab tested, as significantly minimizing panel weight would lead to a dramatic increase in stab penetration depth, compromising protection.

Moreover, it is widely known that the toughness and impact resistance of materials are related. However, in the context of 3D-printed armor, this relationship still requires confirmation due to the nature of stabbing, which involves indentation, penetration, and perforation [59]. The Pearson correlation method revealed a strong correlation between toughness and impact resistance, with an r value of 0.550, which was statistically significant, as confirmed by a *p*-value of 0.003.

**Figure 8:**
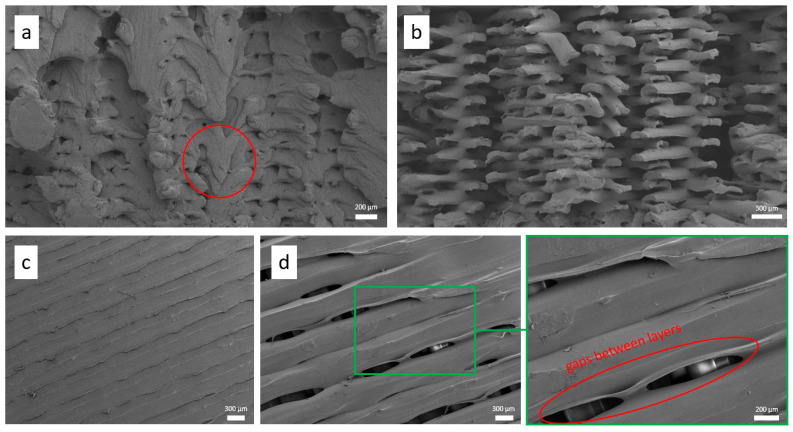
SEM images of representative specimens showing the influence of Id and Lw on porosity and interlayer bonding. (**a**) specimen 7 with 0.1 mm Lt and 100% Id, showing dense morphology and minimal voids, (**b**) specimen 1 with 0.1 mm Lt and 50% Id, revealing larger pores, (**c**) specimen 1 with 0.2 mm Lw, illustrating uniform layer stacking, and (**d**) specimen 27 with 0.3 mm Lw, where visible gaps between layers indicate poor consolidation and higher porosity.

As observed from the statistical analysis, Id was the most significant contributor to all investigated responses. This is because higher Id creates denser structures with fewer voids and internal porosities, improving interlayer bonding [36]. This enhances energy absorption and impact resistance by optimizing load distribution and reducing localized deformation, as more material resists applied loads and impacts. Therefore, the SEM images presented in Figure 8 were analyzed to evaluate the effect of these parameters. As seen in Figure 8a,b, which show specimens 7 and 1 with 100% and 50% Id, respectively, the 100% Id specimen exhibited a structure closer to that of injection molding, with fewer porosities compared to the 50% Id specimen, which showed significant porosity. Both the statistical analysis and morphological investigation confirm that higher Id produces denser parts with fewer internal porosities and stronger interlayer fusion, thereby improving mechanical and protective performance by providing more material to resist impacts. However, increasing Id also affects material consumption and, consequently, increases the overall part weight, highlighting the need for optimization. Future research should therefore focus on balancing protective performance and weight in 3D-printed protective textiles to achieve an optimal trade-off. Moreover, as demonstrated by the red circle in Figure 8a, the increased Pt of specimen 7 (printed at 270 °C) compared to specimen 1 (printed at 250 °C) caused increased material flow due to higher liquidity. This affected internal bonding and homogeneity. Figure 8c,d compare the effect of Lw on specimens 7 and 27, respectively. A lower Lw resulted in a denser structure with minimal gaps between layers, whereas a larger Lw led to significant gaps (highlighted by the red ellipse in Figure 8d) due to insufficient interlayer bonding, negatively impacting the overall performance of these structures.

### 3.2. ANN Results

**Figure 9:**
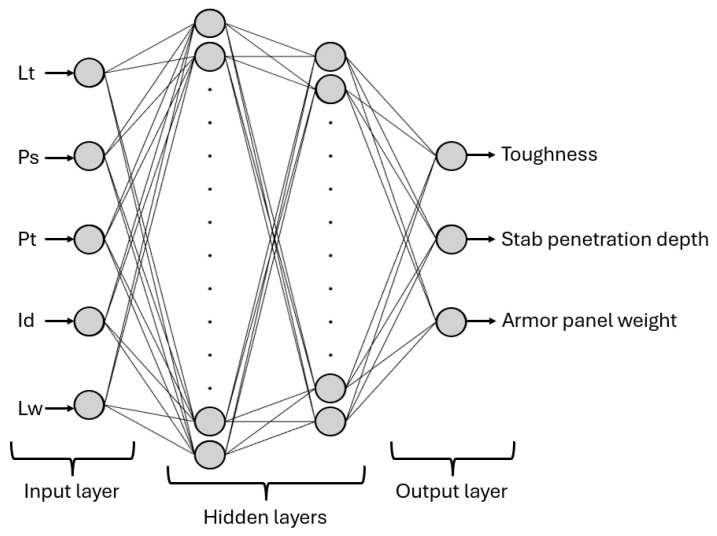
Simplified 5-14-12-3 ANN structure.

Among the tested ANN models with different topologies, as described in Section 2.4, the best performance was obtained with two hidden layers containing 14 and 12 neurons, respectively. This topology was selected based on its minimum performance function value of 0.0061935, which can be considered effectively equivalent to zero. The resulting 5-14-12-3 ANN architecture is illustrated in Figure 9.

**Figure 10:**
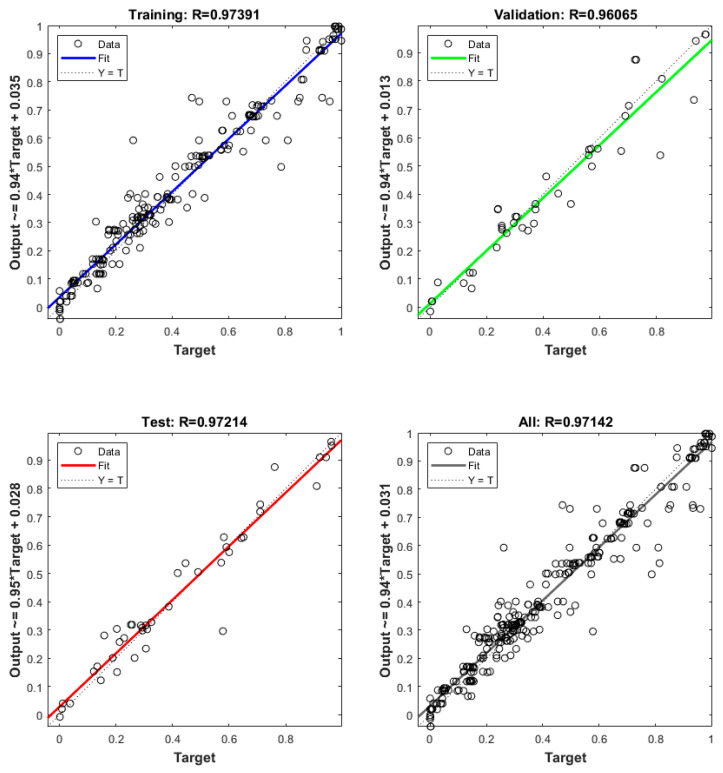
ANN Model Performance: training, validation, and test sets.

Furthermore, the performance of the ANN model, assessed through linear regression, is illustrated in Figure 10. Overall, the model exhibited excellent performance in predicting toughness, stab penetration depth, and armor panel weight, with an R^2^ value of 0.97142. The R^2^ values recorded for training, validation, and testing were 0.97391, 0.96065, and 0.97214, respectively. Additionally, the MSE values for the best-fit ANN structure were 0.0048, 0.0059, and 0.0047 for training, validation, and testing.

**Figure 11:**
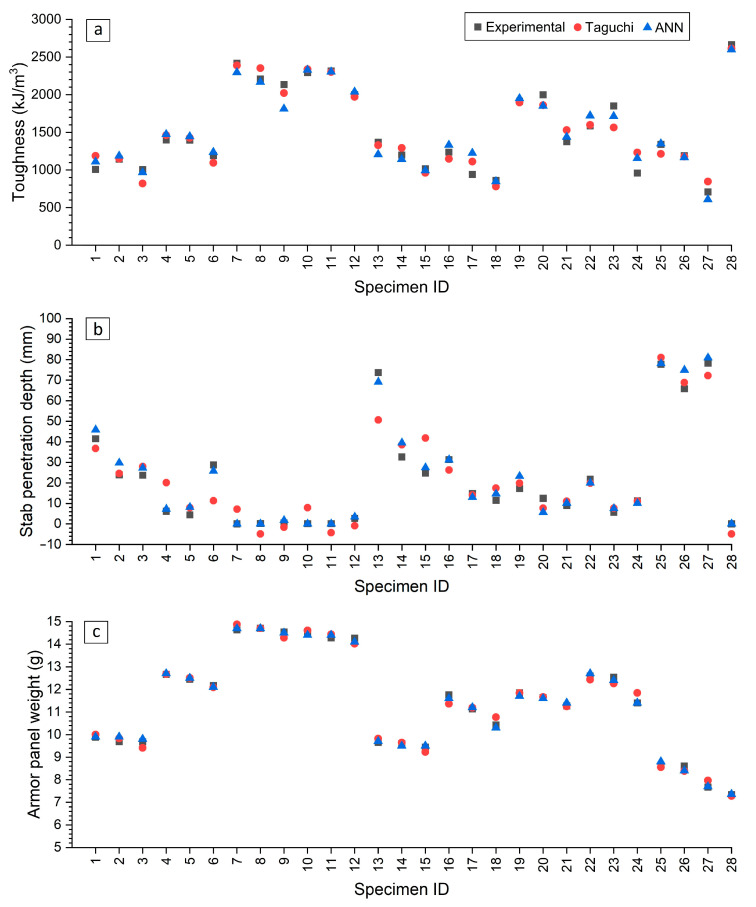
Comparison of the results. (**a**) toughness, (**b**) stab penetration depth, and (**c**) armor panel weight. The best values for each output are represented by specimen ID 28.

To further assess the performance of the developed ANN in predicting the outputs investigated, a comparison with experimental and Taguchi results was conducted by evaluating the percentage error. The results, shown in Figure 11, reveal that the ANN model outperformed the Taguchi method, primarily due to its ability to capture the nonlinear relationships within the process. The ANN model predicted toughness and armor panel weight with similar accuracy as the Taguchi method, with only around 5% error from the experimental data, closely aligning with the experimental results as illustrated in Figure 11a,c. However, the ANN model demonstrated an 8 times better performance in predicting stab penetration depth compared to the Taguchi method, as seen in Figure 11b, where the experimental and ANN results were nearly identical, while the Taguchi results exhibited negative values, which are physically impossible. In summary, the ANN model exhibited similar performance in accurately predicting the results as the Taguchi method. This is because the ANN, being a non-linear data-driven model, was able to represent more complex parameter–response relationships, which explains its superior predictive accuracy. These methods should therefore be seen as complementary: Taguchi provides a structured and efficient experimental framework, while the ANN extends the analysis by modeling non-linearities in the data.

It is important to note that although ANNs are sometimes considered black-box models, in this study, the ANN was used not merely as an evaluation tool but as a predictive tool complementary to Taguchi and statistical analysis. The predictions were compared against experimental outcomes and validated through morphological observations, ensuring consistency with physical mechanisms. Thus, the ANN served as both a predictive and comparative tool in this study, paving the way for future research on its suitability to predict the performance of 3D-printed parts.

## 4. Conclusions

The growing interest in 3D-printed SRBA highlights the need to better understand the relationship between print parameters and structural performance. Therefore, this study has systematically investigated the influence of five different printing parameters on the performance metrics of 3D-printed protective textiles. The Taguchi L_27_ orthogonal array approach and ANOVA results revealed that infill density emerged as the most influential factor across all variables, which was also corroborated by observations from SEM images. Increasing Id enhanced toughness while significantly reducing stab penetration depth. Optimized parameters yielded specimens with zero recorded penetration depth, surpassing the UK HOSDB body armor standard of a permitted knife penetration up to 8 mm. However, while improving protection, increasing Id also increases weight, posing a challenge to overall wearability. Moreover, the developed 5-14-12-3 ANN model demonstrated predictive performance similar to that of the Taguchi approach, validating its potential for modeling and optimization in 3D-printed protective applications. While this study has established a solid foundation for enhancing 3D-printed protective textiles, all experiments were conducted under controlled laboratory conditions, without varying environmental factors such as humidity or temperature. However, these parameters are known to influence the mechanical behavior of polymers. Future work will therefore extend validation to diverse environmental conditions to ensure the robustness and reliability of 3D-printed stab-resistant panels in real-world applications. In addition, further optimization of printing parameters will be required to achieve an optimal balance between protection and weight, supporting the advancement of AM as a practical solution for next-generation lightweight and high-performance protective systems.

## Figures and Tables

**Table 1:** Fixed and variable process parameters.

Parameter		Unit	Value
**Fixed**	Infill type	-	Rectilinear
	Raster angle	°	±45
	Number of perimeters	-	2
	Bed temperature	°C	107
**Variable**	Layer thickness	mm	0.1; 0.2; 0.3
	Print speed	mm/s	30; 40; 50
	Printing temperature	°C	250; 260; 270
	Infill density	%	50; 75; 100
	Layer width	mm	0.2; 0.35; 0.5

**Table 2:** L_27_ Taguchi orthogonal array.

ID	L_27_ Taguchi Design				
	Lt	Ps	Pt	Id	Lw
	mm	mm/s	°C	%	mm
**1**	0.1	30	250	50	0.20
**2**	0.1	30	250	50	0.35
**3**	0.1	30	250	50	0.50
**4**	0.1	40	260	75	0.20
**5**	0.1	40	260	75	0.35
**6**	0.1	40	260	75	0.50
**7**	0.1	50	270	100	0.20
**8**	0.1	50	270	100	0.35
**9**	0.1	50	270	100	0.50
**10**	0.2	30	260	100	0.20
**11**	0.2	30	260	100	0.35
**12**	0.2	30	260	100	0.50
**13**	0.2	40	270	50	0.20
**14**	0.2	40	270	50	0.35
**15**	0.2	40	270	50	0.50
**16**	0.2	50	250	75	0.20
**17**	0.2	50	250	75	0.35
**18**	0.2	50	250	75	0.50
**19**	0.3	30	270	75	0.20
**20**	0.3	30	270	75	0.35
**21**	0.3	30	270	75	0.50
**22**	0.3	40	250	100	0.20
**23**	0.3	40	250	100	0.35
**24**	0.3	40	250	100	0.50
**25**	0.3	50	260	50	0.20
**26**	0.3	50	260	50	0.35
**27**	0.3	50	260	50	0.50

**Table 3:** Experimental results and SN ratios.

ID	Average Results			SN Ratios		
	Toughness	Stab Penetration Depth	Armor Panel Weight	Toughness	Stab Penetration Depth	Armor Panel Weight
	kJ/m^3^	mm	g	dB	dB	dB
**1**	1009.04 ± 35.22	41.50 ± 2.36	9.88 ± 0.03	60.08	−32.37	−19.89
**2**	1144.86 ± 49.01	23.87 ± 2.19	9.69 ± 0.06	61.18	−27.59	−19.73
**3**	1007.32 ± 39.56	23.76 ± 2.09	9.68 ± 0.05	60.06	−27.54	−19.72
**4**	1399.22 ± 60.67	6.20 ± 2.87	12.67 ± 0.08	62.92	−16.54	−22.06
**5**	1396.79 ± 25.46	4.45 ± 0.28	12.45 ± 0.03	62.90	−12.99	−21.90
**6**	1191.58 ± 26.41	28.72 ± 2.85	12.17 ± 0.06	61.52	−29.20	−21.70
**7**	2418.25 ± 46.99	0.02 ± 0.00	14.64 ± 0.04	67.67	33.98	−23.31
**8**	2210.72 ± 66.05	0.19 ± 0.04	14.71 ± 0.05	66.89	14.12	−23.35
**9**	2135.40 ± 40.78	0.46 ± 0.23	14.54 ± 0.06	66.59	5.90	−23.25
**10**	2293.41 ± 79.78	0.09 ± 0.05	14.52 ± 0.07	67.21	19.95	−23.24
**11**	2315.76 ± 86.71	0.06 ± 0.01	14.28 ± 0.07	67.29	25.00	−23.09
**12**	2003.42 ± 38.86	2.66 ± 1.16	14.27 ± 0.07	66.04	−9.13	−23.09
**13**	1368.85 ± 44.70	73.70 ± 2.74	9.65 ± 0.09	62.73	−37.35	−19.69
**14**	1199.72 ± 46.86	32.61 ± 2.63	9.60 ± 0.07	61.58	−30.29	−19.65
**15**	1016.81 ± 64.89	24.68 ± 2.08	9.45 ± 0.04	60.14	−27.87	−19.51
**16**	1237.59 ± 74.19	31.31 ± 7.00	11.76 ± 0.09	61.85	−30.08	−21.41
**17**	941.52 ± 14.07	14.80 ± 2.12	11.14 ± 0.03	59.48	−23.48	−20.94
**18**	862.62 ± 15.76	11.48 ± 1.10	10.43 ± 0.10	58.72	−21.23	−20.36
**19**	1913.65 ± 67.54	17.23 ± 1.65	11.85 ± 0.06	65.64	−24.76	−21.47
**20**	1998.79 ± 84.78	12.45 ± 1.26	11.64 ± 0.09	66.02	−21.94	−21.32
**21**	1376.39 ± 47.20	8.95 ± 2.96	11.27 ± 0.08	62.77	−19.40	−21.04
**22**	1587.01 ± 54.73	21.72 ± 3.21	12.60 ± 0.07	64.01	−26.81	−22.01
**23**	1850.61 ± 62.43	5.71 ± 2.68	12.54 ± 0.03	65.35	−15.84	−21.96
**24**	959.80 ± 19.10	11.22 ± 1.70	11.40 ± 0.09	59.64	−21.08	−21.14
**25**	1341.42 ± 67.46	77.76 ± 1.88	8.63 ± 0.05	62.55	−37.82	−18.72
**26**	1189.84 ± 35.66	65.87 ± 5.93	8.61 ± 0.03	61.51	−36.40	−18.70
**27**	709.86 ± 28.85	78.30 ± 4.96	7.67 ± 0.11	57.02	−37.89	−17.70

**Table 4:** Detailed Taguchi results for toughness.

Level	Lt	Ps	Pt	Id	Lw
**1**	63.12	63.71	61.01	60.45	63.47
**2**	62.51	62.11	62.89	62.14	63.34
**3**	62.29	62.11	64.04	65.34	61.12
**Δ**	0.83	1.60	3.03	4.89	2.35
**Rank**	5	4	2	1	3

**Table 5:** Individual contribution of printing parameters on toughness.

Parameter	DF	Adj SS	Adj MS	F-Value	*p*-Value	Contribution
**Lt**	2	56,405.13	28,203.57	1.10	0.357	0.84%
**Ps**	2	547,534.33	273,767.17	10.68	0.001	8.11%
**Pt**	2	1,448,786.97	724,393.99	28.26	0.000	21.46%
**Id**	2	3,549,388.18	1,774,694.09	69.23	0.000	52.58%
**Lw**	2	738,508.57	369,254.79	14.41	0.000	10.94%
**Error**	16	410,128.99	25,633.00			6.08%
**Total**	26	6,750,749.18				100%

**Table 6:** Detailed Taguchi results for stab penetration depth.

Level	Lt	Ps	Pt	Id	Lw
**1**	−10.25	−13.08	−25.11	−32.80	−16.87
**2**	−14.94	−24.22	−15.00	−22.18	−14.38
**3**	−26.88	−14.77	−11.96	2.90	−20.83
**Δ**	16.63	11.13	13.16	35.69	6.45
**Rank**	2	4	3	1	5

**Table 7:** Individual contribution of printing parameters on stab penetration depth.

Parameter	DF	Adj SS	Adj MS	F-Value	*p*-Value	Contribution
**Lt**	2	1644.53	822.26	7.54	0.005	10.52%
**Ps**	2	1244.98	622.49	5.71	0.013	7.96%
**Pt**	2	564.08	282.04	2.59	0.106	3.61%
**Id**	2	9725.20	4862.60	44.59	0.000	62.20%
**Lw**	2	710.73	355.37	3.26	0.065	4.55%
**Error**	16	1744.82	109.05			11.16%
**Total**	26	15,634.34				100%

**Table 8:** Detailed Taguchi results for armor panel weight.

Level	Lt	Ps	Pt	Id	Lw
**1**	−21.66	−21.40	−20.80	−19.25	−21.31
**2**	−21.22	−21.07	−21.13	−21.36	−21.18
**3**	−20.45	−20.86	−21.40	−22.72	−20.83
**Δ**	1.21	0.54	0.60	3.46	0.48
**Rank**	2	4	3	1	5

**Table 9:** Individual contribution of printing parameters on armor panel weight.

Parameter	DF	Adj SS	Adj MS	F-Value	*p*-Value	Contribution
**Lt**	2	11.45	5.72	80.55	0.000	10.21%
**Ps**	2	1.69	0.84	11.87	0.001	1.50%
**Pt**	2	4.05	2.03	28.52	0.000	3.62%
**Id**	2	92.12	46.060	648.30	0.000	82.17%
**Lw**	2	1.66	0.83	11.70	0.001	1.48%
**Error**	16	1.14	0.07			1.01%
**Total**	26	112.11				100%

## Data Availability

The original contributions presented in this study are included in this article. Further inquiries can be directed to the corresponding author.

## Supplementary Materials

The following supporting information can be downloaded at: https://www.mdpi.com/article/10.3390/polym17192699/s1, Table S1: Detailed experimental results.

## Abbreviations

The following abbreviations are used in this manuscript:

AM	Additive manufacturing
ANN	Artificial neural network
ASTM	American Society for Testing and Materials
PBF	Powder bed fusion
MEX	Material extrusion
SRBA	Stab-resistant body armor
SLS	Selective laser sintering
HOSDB	Home Office Scientific Development Branch
PA	Polyamide
FFF	Fused filament fabrication
PC	Polycarbonate
FEA	Finite element analysis
ABS	Acrylonitrile butadiene styrene
PLA	Polylactic acid
ML	Machine learning
RSM	Response surface methodology
ANOVA	Analysis of variance
Lt	Layer thickness
Ps	Print speed
Pt	Print temperature
Id	Infill density
Lw	Layer width
SN	Signal-to-noise
LtB	Larger-the-better
StB	Smaller-the-better
SEM	Scanning electron microscopy
trainlm	Levenberg–Marquardt algorithm
MSE	Mean squared error
R^2^	Coefficient of determination
