# Supplementary material for: Evaluating the Performance of 3D-Printed Stab-Resistant Body Armor Using the Taguchi Method and Artificial Neural Networks
Source: Polymers (Basel). 2025 Oct 7;17(19):2699. doi: 10.3390/polym17192699 (PMC12526826; doi:10.3390/polym17192699)
Supplement: Supplementary file 1 [file polymers-17-02699-s001.zip › polymers-3895450-supplementary.pdf]

**Supplementary Table S1**. Detailed experimental results.

| Toughness value of each specimen | | | | | | | |
|---|---|---|---|---|---|---|---|
| **Sample ID** | **Specimen** | | | | | **Mean (kJ/m³)** | **SD** |
| | **1** | **2** | **3** | **4** | **5** | | |
| **1** | 960.72 | 984.88 | 1021.12 | 1033.20 | 1045.28 | 1009.04 | 35.22 |
| **2** | 1077.62 | 1111.24 | 1161.67 | 1178.48 | 1195.29 | 1144.86 | 49.01 |
| **3** | 953.04 | 980.18 | 1020.89 | 1034.46 | 1048.03 | 1007.32 | 39.56 |
| **4** | 1315.98 | 1357.60 | 1420.03 | 1440.84 | 1461.65 | 1399.22 | 60.67 |
| **5** | 1361.86 | 1379.32 | 1405.52 | 1414.26 | 1422.99 | 1396.79 | 25.46 |
| **6** | 1155.35 | 1173.46 | 1200.64 | 1209.70 | 1218.76 | 1191.58 | 26.41 |
| **7** | 2353.78 | 2386.02 | 2434.37 | 2450.48 | 2466.60 | 2418.25 | 46.99 |
| **8** | 2120.10 | 2165.41 | 2233.37 | 2256.03 | 2278.68 | 2210.72 | 66.05 |
| **9** | 2079.45 | 2107.43 | 2149.39 | 2163.37 | 2177.36 | 2135.40 | 40.78 |
| **10** | 2183.95 | 2238.68 | 2320.77 | 2348.14 | 2375.50 | 2293.41 | 79.78 |
| **11** | 2196.79 | 2256.28 | 2345.50 | 2375.24 | 2404.98 | 2315.76 | 86.71 |
| **12** | 1950.10 | 1976.76 | 2016.75 | 2030.08 | 2043.41 | 2003.42 | 38.86 |
| **13** | 1307.52 | 1338.19 | 1384.18 | 1399.51 | 1414.85 | 1368.85 | 44.70 |
| **14** | 1135.43 | 1167.57 | 1215.79 | 1231.87 | 1247.94 | 1199.72 | 46.86 |
| **15** | 927.78 | 972.30 | 1039.07 | 1061.32 | 1083.58 | 1016.81 | 64.89 |
| **16** | 1135.80 | 1186.70 | 1263.04 | 1288.48 | 1313.93 | 1237.59 | 74.19 |
| **17** | 922.22 | 931.87 | 946.35 | 951.17 | 956.00 | 941.52 | 14.07 |
| **18** | 841.00 | 851.81 | 868.03 | 873.43 | 878.84 | 862.62 | 15.76 |
| **19** | 1820.99 | 1867.32 | 1936.82 | 1959.98 | 1983.15 | 1913.65 | 67.54 |
| **20** | 1882.47 | 1940.63 | 2027.87 | 2056.95 | 2086.03 | 1998.79 | 84.78 |
| **21** | 1311.63 | 1344.01 | 1392.58 | 1408.77 | 1424.96 | 1376.39 | 47.20 |
| **22** | 1511.92 | 1549.47 | 1605.78 | 1624.55 | 1643.33 | 1587.01 | 54.73 |
| **23** | 1764.96 | 1807.78 | 1872.02 | 1893.44 | 1914.85 | 1850.61 | 62.43 |
| **24** | 933.60 | 946.70 | 966.35 | 972.90 | 979.45 | 959.80 | 19.10 |
| **25** | 1248.87 | 1295.14 | 1364.56 | 1387.70 | 1410.84 | 1341.42 | 67.46 |
| **26** | 1140.91 | 1165.38 | 1202.07 | 1214.30 | 1226.53 | 1189.84 | 35.66 |
| **27** | 670.28 | 690.07 | 719.76 | 729.65 | 739.55 | 709.86 | 28.85 |
| **Stab penetration depth of each specimen** | | | | | | | |
| **Sample ID** | **Specimen** | | | | | **Mean (mm)** | **SD** |
| | **1** | **2** | **3** | **4** | **5** | | |
| **1** | 42.52 | 43.52 | 40.00 | 43.35 | 38.10 | 41.50 | 2.36 |
| **2** | 23.87 | 24.10 | 20.54 | 24.16 | 26.69 | 23.87 | 2.19 |
| **3** | 21.54 | 26.08 | 23.53 | 25.70 | 21.95 | 23.76 | 2.09 |
| **4** | 10.16 | 4.37 | 8.32 | 3.58 | 4.57 | 6.20 | 2.87 |
| **5** | 4.14 | 4.41 | 4.90 | 4.43 | 4.39 | 4.45 | 0.28 |
| **6** | 28.32 | 26.15 | 29.41 | 26.51 | 33.23 | 28.72 | 2.85 |
| **7** | 0.02 | 0.02 | 0.02 | 0.02 | 0.02 | 0.02 | 0.00 |
| **8** | 0.15 | 0.20 | 0.18 | 0.19 | 0.25 | 0.19 | 0.04 |
| **9** | 0.72 | 0.32 | 0.64 | 0.48 | 0.16 | 0.46 | 0.23 |
| **10** | 0.10 | 0.09 | 0.10 | 0.00 | 0.15 | 0.09 | 0.05 |
| **11** | 0.05 | 0.06 | 0.05 | 0.06 | 0.06 | 0.06 | 0.01 |
| **12** | 0.96 | 2.00 | 3.36 | 3.80 | 3.20 | 2.66 | 1.16 |
| **13** | 73.42 | 73.22 | 70.09 | 74.01 | 77.78 | 73.70 | 2.74 |
| **14** | 32.94 | 29.47 | 30.37 | 35.12 | 35.14 | 32.61 | 2.63 |

| | | | | | | | |
|---|---|---|---|---|---|---|---|
| **15** | 23.85 | 23.91 | 27.61 | 22.20 | 25.82 | 24.68 | 2.08 |
| **16** | 31.15 | 31.88 | 42.59 | 25.70 | 25.22 | 31.31 | 7.00 |
| **17** | 12.46 | 13.87 | 16.68 | 17.39 | 13.62 | 14.80 | 2.12 |
| **18** | 11.40 | 11.90 | 13.02 | 11.07 | 10.03 | 11.48 | 1.10 |
| **19** | 17.10 | 17.85 | 19.53 | 16.61 | 15.05 | 17.23 | 1.65 |
| **20** | 11.48 | 12.20 | 11.47 | 12.57 | 14.53 | 12.45 | 1.26 |
| **21** | 5.60 | 7.91 | 7.12 | 12.20 | 11.93 | 8.95 | 2.96 |
| **22** | 21.70 | 21.00 | 26.12 | 22.58 | 17.19 | 21.72 | 3.21 |
| **23** | 3.96 | 5.56 | 8.00 | 2.34 | 8.70 | 5.71 | 2.68 |
| **24** | 11.19 | 12.54 | 13.25 | 9.91 | 9.21 | 11.22 | 1.70 |
| **25** | 76.93 | 79.44 | 78.72 | 74.85 | 78.87 | 77.76 | 1.88 |
| **26** | 76.25 | 65.07 | 63.74 | 62.35 | 61.94 | 65.87 | 5.93 |
| **27** | 75.00 | 85.50 | 74.50 | 75.00 | 81.50 | 78.30 | 4.96 |
| **Armor panel weight of each specimen** | | | | | | | |
| **Sample ID** | **Specimen** | | | | | **Mean (g)** | **SD** |
| | **1** | **2** | **3** | **4** | **5** | | |
| **1** | 9.88 | 9.86 | 9.83 | 9.90 | 9.91 | 9.88 | 0.03 |
| **2** | 9.73 | 9.65 | 9.78 | 9.66 | 9.65 | 9.69 | 0.06 |
| **3** | 9.77 | 9.66 | 9.68 | 9.64 | 9.64 | 9.68 | 0.05 |
| **4** | 12.63 | 12.61 | 12.61 | 12.78 | 12.72 | 12.67 | 0.08 |
| **5** | 12.46 | 12.44 | 12.41 | 12.44 | 12.48 | 12.45 | 0.03 |
| **6** | 12.19 | 12.11 | 12.23 | 12.20 | 12.10 | 12.17 | 0.06 |
| **7** | 14.60 | 14.61 | 14.64 | 14.62 | 14.71 | 14.64 | 0.04 |
| **8** | 14.68 | 14.77 | 14.64 | 14.69 | 14.75 | 14.71 | 0.05 |
| **9** | 14.49 | 14.57 | 14.59 | 14.58 | 14.45 | 14.54 | 0.06 |
| **10** | 14.46 | 14.55 | 14.50 | 14.62 | 14.47 | 14.52 | 0.07 |
| **11** | 14.30 | 14.34 | 14.34 | 14.17 | 14.25 | 14.28 | 0.07 |
| **12** | 14.36 | 14.34 | 14.21 | 14.26 | 14.20 | 14.27 | 0.07 |
| **13** | 9.52 | 9.59 | 9.70 | 9.74 | 9.68 | 9.65 | 0.09 |
| **14** | 9.61 | 9.70 | 9.62 | 9.52 | 9.55 | 9.60 | 0.07 |
| **15** | 9.51 | 9.44 | 9.42 | 9.48 | 9.40 | 9.45 | 0.04 |
| **16** | 11.69 | 11.84 | 11.67 | 11.74 | 11.87 | 11.76 | 0.09 |
| **17** | 11.12 | 11.17 | 11.17 | 11.10 | 11.14 | 11.14 | 0.03 |
| **18** | 10.35 | 10.37 | 10.39 | 10.43 | 10.59 | 10.43 | 0.10 |
| **19** | 11.78 | 11.78 | 11.89 | 11.90 | 11.90 | 11.85 | 0.06 |
| **20** | 11.70 | 11.72 | 11.64 | 11.62 | 11.50 | 11.64 | 0.09 |
| **21** | 11.41 | 11.27 | 11.22 | 11.22 | 11.22 | 11.27 | 0.08 |
| **22** | 12.60 | 12.50 | 12.68 | 12.59 | 12.65 | 12.60 | 0.07 |
| **23** | 12.59 | 12.50 | 12.52 | 12.54 | 12.53 | 12.54 | 0.03 |
| **24** | 11.37 | 11.28 | 11.40 | 11.51 | 11.45 | 11.40 | 0.09 |
| **25** | 8.58 | 8.60 | 8.59 | 8.66 | 8.70 | 8.63 | 0.05 |
| **26** | 8.59 | 8.62 | 8.59 | 8.66 | 8.58 | 8.61 | 0.03 |
| **27** | 7.57 | 7.75 | 7.67 | 7.81 | 7.57 | 7.67 | 0.11 |
